# Prize Essays

**Published:** 1867-06

**Authors:** 


PRIZE ESSAYS.

Dr. F. Donaldson, of Baltimore, from the Committee on Prize Essays, reported that they had received eight essays, all of which had been carefully examined, and after fully weighing the merits of each, two had been agreed upon as being the most meritorious, and they had awarded the prize of $100 each to the following:

First—"An essay on the Causes of Intermittent and Remittent Fever," bearing the motto, "Fortes est veritas."

Second—"On the Treatment of Certain Abnormities of the Uterus," Motto, "Empyricism in Medicine and Surgery is fast giving way to the Rationalism of true Diagnosis."

The sealed envelopes containing the names of the authors were then opened, and the announcement made that the author of the first is Dr. J. D. Black, of Newark, Ohio; of the second, Dr. Montrose A. Pallen, of St. Louis, Mo.

